# Improving Preschoolers’ Arithmetic through Number Magnitude Training: The Impact of Non-Symbolic and Symbolic Training

**DOI:** 10.1371/journal.pone.0166685

**Published:** 2016-11-22

**Authors:** Nastasya Honoré, Marie-Pascale Noël

**Affiliations:** Psychological Sciences Research Institute, Université Catholique de Louvain, Louyain-la-Neuve, Belgium; Universiteit Gent, BELGIUM

## Abstract

The numerical cognition literature offers two views to explain numerical and arithmetical development. The unique-representation view considers the approximate number system (ANS) to represent the magnitude of both symbolic and non-symbolic numbers and to be the basis of numerical learning. In contrast, the dual-representation view suggests that symbolic and non-symbolic skills rely on different magnitude representations and that it is the ability to build an exact representation of symbolic numbers that underlies math learning. Support for these hypotheses has come mainly from correlative studies with inconsistent results. In this study, we developed two training programs aiming at enhancing the magnitude processing of either non-symbolic numbers or symbolic numbers and compared their effects on arithmetic skills. Fifty-six preschoolers were randomly assigned to one of three 10-session-training conditions: (1) non-symbolic training (2) symbolic training and (3) control training working on story understanding. Both numerical training conditions were significantly more efficient than the control condition in improving magnitude processing. Moreover, symbolic training led to a significantly larger improvement in arithmetic than did non-symbolic training and the control condition. These results support the dual-representation view.

## Introduction

**Fig 1:**
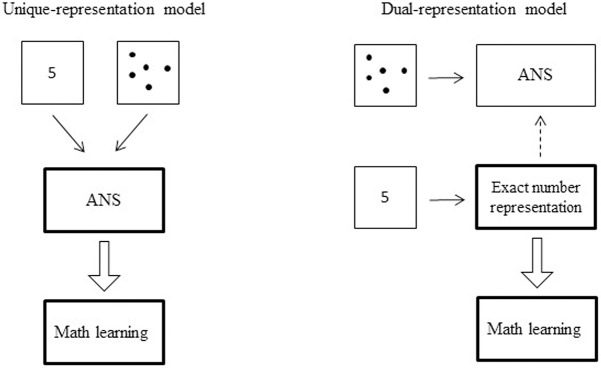
Schematic illustration of the numerical development views. In the unique-representation view, the ANS is the basis of mathematics and is activated by both symbolic and non-symbolic numbers. In the dual-representation view, non-symbolic numbers activate the ANS and symbolic numbers activate an exact representation of numbers; the latter is the root of mathematical learning and can refine the ANS.

Numerical magnitude representation is thought to provide a foundation for higher-level mathematical skills such as calculation. However, the nature of the numerical magnitude underlying this development is a matter of debate which can be summarized into two views (Fig 1, see also [1]).

The unique-representation view considers the Approximate Number System (ANS) to be the root of numerical and arithmetical skills (see [2–3] for a review). This number sense, which is already present in infants (e.g. [4, 5–7]) and shared with non-human animals [8, 9], corresponds to the capacity to nonverbally represent number magnitude by producing imprecise, noisy number magnitude representations [10–12]. The ANS is gradually refined during infancy and school-age years [13] and its precision can be measured in a simple task in which one has to judge which of two arrays of items is more numerous. Performance in this type of task depends on the ratio between the numerosities to discriminate, e.g., it is easier to discriminate 7 from 14 or 8 from 4 (ratio 1:2) than 7 from 8 or 16 from 14 (ratio 7:8). It has been shown that six-month-old infants are able to discriminate between two sets of items differing by a ratio of 1:2 [14] and this threshold ratio increases with age to reach approximately 10:11 at adulthood [15, 16]. According to this view, both symbolic and non-symbolic magnitude comparisons show ratio effects [11, 17, 18] and similar brain areas would be activated for both types of stimuli ([19–21], see [2, 17] for a review). These studies suggest that symbolic and non-symbolic magnitude processing activate one approximate magnitude representation system, the ANS [22]. Finally, the unique-representation view postulates that the ANS underlies math learning [13, 23] as ANS acuity has been shown to correlate with individual differences in mathematics competences [15, 24] and to be lower in people presenting specific math learning disability or dyscalculia [25–27].

In contrast, the dual-representation view considers that symbolic and non-symbolic numbers activate distinct and non-overlapping representations (see [1]). Indeed, symbolic and non-symbolic magnitude processing skills have distinct developmental trajectories [28]; symbolic and non-symbolic distance effects do not correlate [29–33] and different processes generate symbolic and non-symbolic ratio effects [34]. In addition, recent brain imaging studies using more precise techniques failed to find overlapping representations between dots and digits [35]. According to this view, dot sets activate an analogue and approximate representation such as the ANS [22]. However, recent studies question the extent to which this representation would really code for number magnitude [36]. Indeed, as it is impossible to perfectly control for non-numerical parameters [37], the tasks tapping the ANS also measure the ability to process other physical parameters covarying with numerosity and inhibitory control [38]. As regards symbolic numbers, they provide access to a more precise number representation (e.g. [39, 40]). Indeed, symbolic numbers would not acquire their meaning through a mapping with the ANS but through the development of a new representation. According to Carey’s developmental view [41–43], the understanding of exact natural numbers is built on a parallel individuation system allowing babies to keep track of the items in a small set. Then the child discovers the successor function through the analogy between the order of the number words in the counting list and the quantities in a series of sets related by additional elements. Furthermore, it has been proposed that symbolic number representations are place-coded, i.e. an Arabic number activates its corresponding representation with a maximal strength but also activates surrounding number representations, with a gradually decreasing strength; whereas non-symbolic numerosities are summation-coded, i.e. a dot set activates its target quantity but also includes all numbers representations up to the target [44, 45]. According to this dual-representation view, mathematical learning relies on the ability to process symbolic numbers’ magnitude. Indeed, the absence of words for numbers prevents access to simple exact arithmetic despite the presence of analogue estimation abilities [16, 46] and several studies have shown that mathematics achievement is related to performance in symbolic magnitude comparison, but not in non-symbolic magnitude comparison tasks ([47], see [48, 49, 50, 51] for a review] and that dyscalculia emerges from a deficit in the magnitude representation of symbolic numbers [1, 52] rather than from a deficit of the ANS. But this dual-representation view does not reject the role of the ANS. For instance, Le Corre and Carey [53] assume that, although the ANS has a quite limited role in the acquisition process of the counting list, once children have built the exact representation of symbolic numbers, they come to establish connections between these new representations and the ANS. Similarly, Piazza, Pica, Izard, Spelke and Dehaene [54] demonstrated, in Mundurucù participants (an Amazonian ethnic group whose language lacks number words beyond 5), that the learning of symbolic numbers and basic arithmetic enhances the acuity of the ANS.

In this study, we wanted to address the question of which number magnitude representation underlies math learning by using a training design. More specifically, we tested whether training the number magnitude representation of non-symbolic or symbolic numbers has an impact on numerical development and on arithmetic in particular. A number of previous studies have already measured the effect of numerical training on numerical development and arithmetic. Numerous authors have demonstrated the efficiency of training games involving magnitude comparison tasks [55–62] or number lines [62–66] and have observed training-related improvement. Although these studies show that it is possible to enhance number magnitude processing, they did not distinguish between training symbolic and non-symbolic number magnitude processing. Furthermore, the few studies which have shown an impact of training on calculation actually included addition or subtraction exercises in the training program itself [57, 58, 63, 65–67]. It is therefore still unknown whether training number magnitude processing alone has an impact on arithmetical competences.

The present study aimed to contrast the predictions based on the unique ANS representation view and the dual symbolic / non-symbolic representations view by comparing the effects of two very similar versions of numerical games, one using symbolic quantities and one using non-symbolic quantities, and comparing the impact of both on numerical and arithmetical competences. Accordingly, we will measure the processing of symbolic and non-symbolic number magnitude and arithmetic before and after training (pre- and post-testing).

According to the unique representation view, both training conditions should yield similar improvement in number magnitude processing tasks, as both symbolic and non-symbolic magnitude processing involve the ANS. To the contrary, the dual-representation view predicts a differential impact of non-symbolic and symbolic training; the former yielding enhancement in non-symbolic processing only and the latter mainly in symbolic processing. However, as working with symbolic numbers has been shown to increase the acuity of the ANS [54], an impact of the symbolic condition on the ANS acuity could also be expected. Finally, and more importantly, we tested whether these types of training, which only involved number magnitude processing, have any significant impact on calculation and examined which type of training has the largest impact on arithmetic. On the one hand, the unique ANS representation view would be expected to provide a similar increase in performance in the two training conditions, as both are assumed to train the ANS, which is supposed to support math learning. On the other hand, according to the dual-representation view, a superior effect of the symbolic condition should be observed because it is the ability to exactly represent numbers, through the learning of the meaning of symbolic numbers, which is associated with mathematics.

## Method

### Participants

The participants were 56 preschool children (91% of contacted children), 27 girls and 29 boys, (mean age = 5 years 9 months, sd = 3.79 months); all but one (Asian) were Caucasian. They were all in their last (third) year of preschool and had not yet received formal mathematics instruction. They had just been introduced at school to counting games and activities involving the recognition and manipulation of numbers from 0 to 9 (e.g. modelling the shapes of the digits with clay). They were recruited from two middle/high social class schools in Walloon Brabant (the French-speaking part of Belgium) and all parents gave their written informed consent. All the children were French-native speakers, apart from one who spoke Portuguese at home, and all were fluent in French. Two others were bilingual (French/English and French/Chinese). The study was approved by the ethics committee of the Psychological Sciences Research Institute.

### Procedure

The study took place at school during school hours for a period of ten weeks. Within each school, participants were randomly divided into three groups: the non-symbolic group (19 children) received the non-symbolic version of numerical training; the symbolic group (19 children) received the symbolic version; and a control group (18 children) received a story-understanding stimulation.

Children were pre-tested during the first two weeks, completed their training program during the next six weeks and were post-tested during the last two weeks. The training program consisted of ten 30-minute sessions implemented in groups of 3 children (groups of 5 children for the control group).

All computerized tasks were created with the E-Prime experimental software (Version 2.0, Psychology Software Tools, Inc., Pittsburgh) and were carried out on a tablet PC with a touch screen.

### Pre- and post-testing

Six tasks were developed: three of them were used to assess the processing of symbolic numbers (Arabic-digit and spoken-word comparison and number line with Arabic numbers), two others evaluated non-symbolic magnitude processing (dot collection comparison and number line with dot sets); and the last one was an exact calculation task.

The pre- and post-measurement tasks were presented in the following order to balance difficulty and maintain motivation: Arabic number comparison—exact additions—non-symbolic number line—collection comparison—verbal number comparison—symbolic number line.

All the tasks, apart from the number-line tasks, were computerized with participants giving their answer by touching the screen with their finger on the side of the correct response. As there is so much variability among reaction times with young children (they stop in the middle of the task, talk when they should answer as fast as possible, look through the window,…) and because a technical problem occurred (the touch screen was not sensitive enough, requiring the participant or the experimenter to touch it several times before it recorded the answer), reaction times were considered as not representative and therefore only accuracy was analyzed.

**1. Collection comparison (non-symbolic):** We adapted Rouselle, Dembour and Noël [68]’s task in which participants were asked to help Dora find the most puzzle pieces. Children were presented with two boxes containing pieces of puzzle (of various sizes) and asked to select the box containing the larger set of puzzle pieces. First a fixation cross appeared in each box; once the child was attentive, the experimenter pressed the space bar and the two collections were simultaneously displayed on both sides of the screen. To prevent children from counting, the collections disappeared after 2000 ms. Fixation crosses appeared again with a question mark until the child answered. To prevent children from relying on non-numerical parameters, perceptual variables were controlled (see [68] for details). First, the external perimeter was equated for all trials. Second, congruent and incongruent trials were built (half of each). In congruent trials, the larger collection in number also had the larger density and surface, whereas in incongruent trials, the larger collection in number had the smaller density and surface. Both collections of a pair had the same smallest and the same largest puzzle piece.

The number of puzzle pieces varied from 5 to 18, i.e. above the subitizing range (1–4) which represents the quantity of items that humans are able to make fast and errorless estimations and that does not rely on analog numerical representation, hence not related to the ANS (see [69] for a review). Six practice pairs of sets differing by a ratio of 1:3 familiarized children with the task. The test trials were pairs of sets differing by ratios of 1:2, 2:3, 3:4, 4:5. There were two pairs per ratio (7–14 and 8–16; 6–9 and 10–15; 6–8 and 12–16; 8–10 and 12–15) and each pair was presented four times, varying along order (ascending or descending) and condition (congruent or incongruent), resulting in 32 items. Items were presented in a fixed random order, respecting five criteria: maximum 3 consecutive same-answer items, maximum 3 consecutive same-condition items, maximum 2 consecutive same-ratio items, no consecutive items of identical pair and the two first pairs were 1:2-ratio items. The percentage of correct responses was calculated and used as the dependent variable. In addition, the effect of ratio was analyzed.

**2. Dot sets on a number line (non-symbolic):** Children were asked to place 18 dot collections (between 2 and 19), without counting them, on a horizontal non-graduated number line (24 cm) presented horizontally in the middle of a half A4 page and marked with one dot and twenty dots on the left and right extremities respectively. A blank line was presented for each item. To prevent the child from relying on non-numerical parameters in the non- symbolic version, the perimeter and size of the dots were manipulated with Matlab to create two conditions: for even numerosities, the external perimeter of the collection-to-position was congruent (positively correlated) and the surface occupied by dots was incongruent (negatively correlated) compared to the right-end collection; for odd numerosities, the perimeter of the collection-to-position was incongruent and the occupied surface was congruent compared to the right-end collection. Each collection contained the same smallest and largest dots.

To measure the child’s precision, we calculated the Percent Absolute Error ($PAE=\left|\frac{estimate-correct\;response}{scale\;of\;estimates}\times 100\right|$). This was used as the dependent variable. We also computed the linear and the logarithmic R^2^ of the best-fitting equation for each participant in order to examine the degree of linearity of estimates, but these measures correlated highly with the precision measure (*r*(56) = -.68, *p* <.001 for lin R^2^ and *r*(56) = -.69, *p* <.001 for log R^2^). Therefore, only the analyses of the precision measure are reported here. The effect of size (small numbers from 2 to 9 and large numbers from 10 to 19) was also examined.

**3. Arabic number comparison (symbolic):** Children were asked to help Sponge Bob by telling him which of two Arabic numbers (from 1 to 19; 20 was excluded because of its physical dissimilarity to other numbers), presented simultaneously on the screen, was the larger. The trial started with two boxes, one on the left and one on the right side of the screen, each containing a fixation cross. When the participant was attentive, the experimenter pressed the space bar to make the numbers appear in each box and the participant was asked to select the larger one by touching the corresponding box on the screen. The items were displayed until the child answered.

Stimuli varied along two numerical sizes, small (1–9) and large (10–19), and two numerical distances, close (1) and far (3), resulting in four conditions (far-small, close-small, far-large, close-large), each containing 6 different pairs, presented in two orders (ascending and descending) for a total of 48 items (6 pairs x 2 orders x 4 conditions). All the stimuli were presented in a fixed random order, according to four criteria: maximum 3 consecutive same-answer items, maximum 2 consecutive same-condition items, no consecutive items of identical pair. The two first items corresponded to far-small pairs. The percentage of correct responses was used as the dependent variable and the effects of size and distance were analyzed.

**4. Verbal number comparison (symbolic):** This task is similar to the Arabic number comparison task, except that numbers were presented orally and sequentially. The scenario was to help Scrat get lots of nuts by choosing the box containing the most. Two boxes, each containing a fixation cross, were first presented to the participant; when he/she was ready, the left box was highlighted while the first number was orally presented (500 ms) on two speakers set on the left and right of the child. After a pause (500 ms), the right box was highlighted with the oral presentation of the second number (500 ms) on both sides again; finally a question mark appeared in each box to invite the child to answer.

**5. Arabic digits on a number line (symbolic):** Children had to position all the Arabic numbers from 2 to 19 on a horizontal non-graduated number line marked with 1 and 20 (in Arabic format) on the left and right sides. The PAE was used as the dependent variable (the lin and log R^2^ were calculated and also highly correlated with PAE, *r*(56) = -.83, *p* <.001 and *r*(56) = -.83, *p* <.001,so they were not further considered in order to avoid redundant analyses).

For both the non-symbolic and symbolic versions, items were presented in a fixed random order, respecting two criteria: maximum 2 consecutive same-condition items and the first two items were small numerosities.

**Fig 2:**
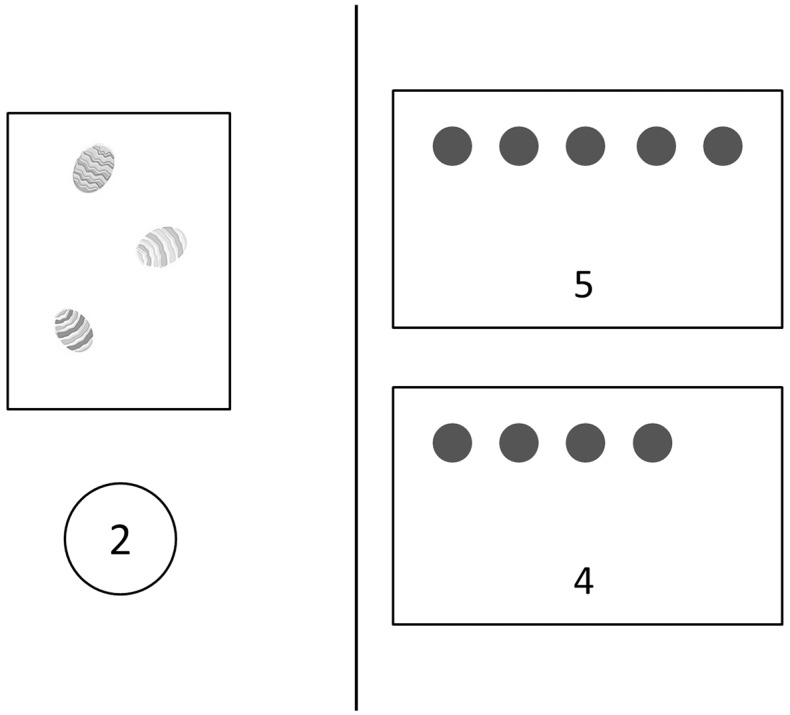
Exact addition task.

**6. Exact additions (symbolic and non-symbolic):** A mixed presentation using both symbolic and non-symbolic formats was chosen (see Fig 2). If only non-symbolic numerosities were used, participants would have simply counted the items and the performance would not have reflected the ability to solve additions but to count stimuli; and if only Arabic numbers were used, it would have advantaged the symbolic group.

Participants were presented with a box containing Easter eggs on the left side of the screen and with an Arabic digit below the egg box. Children were told *“I have some eggs”* while the experimenter pointed at the egg box. Then he stated *“I find [n] more”*, while pointing at the Arabic number corresponding to the amount of added egg(s). On the right side of the screen, there were two boxes, containing either the correct answer or the correct answer +1/-1, presented both in Arabic digits and in sets of dots. Children had to answer the question *“How many Easter eggs do I have now*?*”* by touching the corresponding part of the screen. After 4 practice trials, children were presented 12 test trials. There were two difficulty levels (small (4–6) and large (7–9) sums) each containing 3 different additions presented in both orders (large operand on the top as a set or below as an Arabic digit), resulting in 6 items per difficulty level. The percentage of correct responses was used as the dependent variable and the effect of difficulty level was analyzed.

### Training programs

**1. Numerical training:** To increase the chance of boosting participants’ numerical competences and to avoid boredom, we used two games in each training program. One game was based on number comparison and the other on number line positioning as these tasks have been used in previous programs aiming at enhancing numerical development and have been proven to be effective [55, 57–61, 63, 64, 66]. Therefore, each training condition consisted of two tasks: comparison of dot collections and positioning of dot sets on a number line in the non-symbolic condition; comparison of Arabic numbers and Arabic numbers positioning on a number line for the symbolic condition.

Both games included several levels of difficulty: children started with the easiest level and more difficult levels were presented when the child performed successfully (85% correct responses) at the current level. Feedback was provided after each response. The training programs were computerized and each child played alone on his own computer.

**Fig 3:**
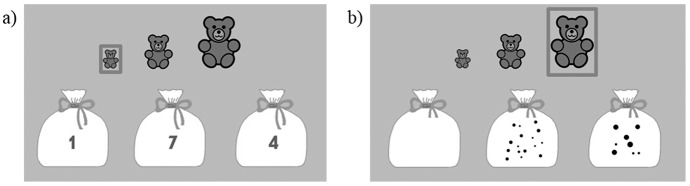
Comparison game. Illustration of the symbolic (a) and the non-symbolic (b) versions.

In the comparison game, children were presented with triplets of Arabic numbers/dot collections, displayed on three aligned bags, and with three aligned animals of increasing size (small, medium and big, ordered from left to right). They were asked to give the bag containing the smallest amount of sweets (or balls of wool, seeds, etc. according to the level, represented by the Arabic number/dot collection displayed on the bag) to the smallest bear (or cat, bird, etc. depending on the level) by touching the corresponding bag. Feedback was then given. Next, they had to give a bag to the tallest bear by selecting the one containing the most sweets among the two remaining bags (Fig 3). Again, feedback was provided. For feedback, the character of the current level appeared either on a green screen saying *“Bravo*!*”* or on a red screen and said *“This is not right*, *try again*!*”*. The child was then allowed to try again until he/she succeeded. If he/she did not respond within the allowed time, the character also appeared on a red screen and said *“You have not given your answer*, *try again*!*”*.

In the symbolic version, stimuli varied along two numerical sizes, small (1–9) and large (10–19), and three distances, 3 or 4, 2 and 1, resulting in six levels (small-distance 3 or 4, small-distance 2, small-distance 1, large-distance 3 or 4, large-distance 2, large-distance 1). Each triplet was presented six times (twice in each order: Large-Medium-Small (LMS), Small-Large-Medium (SLM) and Medium-Small-Large (MSL)).

The non-symbolic version of the game was also divided into six levels, depending on the ratio (level 1 = 0.5–0.6; level 2 = 0.62–0.71; level 3 = 0.71–0.77; level 4 = 0.77–0.81; level 5 = 0.82–0.87; level 6 = 0.86–0.89). To prevent children from relying on non-numerical variables in the non-symbolic version, the external perimeter, the size of the dots and the total surface occupied by the dots were manipulated with Matlab. Items are congruent/incongruent according to (1) the external perimeter when the larger numerosity has a 2/3-larger/smaller perimeter and (2) the surface occupied by dots when the larger collection in number has a 2/3-larger/smaller occupied surface. Two types of each pair composing the triplets were therefore created: (1) one pair was congruent concerning the external perimeter and incongruent concerning the occupied surface and (2) one pair was incongruent concerning the external perimeter and congruent concerning the occupied area. Each triplet was presented in two conditions: (a) the smallest pair in congruency 1 and the largest pair in congruency 2 and (b) the smallest pair in congruency 2 and the largest in congruency 1. Each triplet was presented in the three orders. As items varied along three orders and two conditions, each triplet was presented six times (3 orders x 2 conditions (according to congruency): LMS condition A, SLM condition A, MSL condition A, LMS condition B, SLM condition B and MSL condition B).

Inside each level, six blocks were constituted, each containing one presentation of all triplets so that each block was constituted of one third of each order (and a half of each condition in the non- symbolic training). Triplets were displayed for a maximum of 12000ms. To prevent children from counting in the non-symbolic version, a mask appeared, for a duration of 500ms, every 2000ms (i.e. the stimulus was fully observable during 2000ms then partly hidden for 500ms, then fully visible for 2000ms and then partly covered for 500ms and so on). However, the participant could give his/her answer whenever he wanted within this 12000ms period and once he had done so, the stimulus disappeared. The same procedure was used in the symbolic version to make the two programs more comparable.

**Fig 4:**
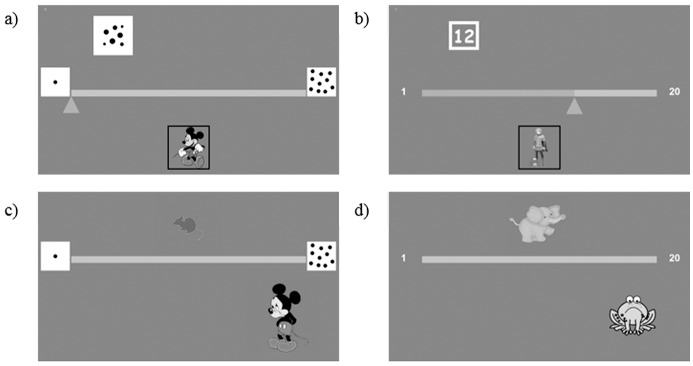
Number line game. Illustration of the non-symbolic (a and c) and symbolic (b and d) versions. In the first level, children were asked to position a numerosity on a number line from 1 to 10 (a) and, in the second level, on a number line from 1 to 20 (b). Feedback was given after each trial; a mouse looking at the left (c) / an elephant looking at the right indicated whether their estimate was too big/small.

In the number-line game, participants were asked to position an Arabic digit (symbolic version) or a dot collection (non-symbolic version) on a number line. The game consisted of helping Mickey Mouse to find Minnie in the first level and a prince to free his princess in the second level by making them move with a given number of steps (indicated in Arabic numbers/dots above the line). At the start of each trial, a cursor was positioned at the left-end of the line, marked with the Arabic digit 1 or with one dot, and children had to slide the cursor with their finger on the screen from that initial position until the target position was reached (Fig 4). While sliding the cursor towards the right side, the line progressively turned blue. In the first level, they had to position an Arabic digit/dot collection between 2 and 9 on a non-graduated number line marked with 1 and 10 on the left and right extremities, respectively (either in Arabic digits for the symbolic version or dot collections for the non-symbolic version). In the second level, they had to place Arabic numbers or dot sets between 2 and 19 on a number line marked 1 (dot) and 20 (dots) on each extremity. Each level was divided into two sub-levels. In the first sub-level, a margin of error of 20% was accepted whereas in the second sub-level the accepted margin of error was reduced to 10%.

To prevent children from relying on non-numerical variables in the non-symbolic version, perceptual variables were controlled. The perimeter and size of the dots were manipulated to create two conditions: (a) the external perimeter of the collection-to-position was congruent with numerosity and the surface occupied by dots was incongruent compared to the right-end collection; (b) the perimeter of the collection-to-position was incongruent with numerosity and the occupied surface was congruent compared to the right-end collection. Children were instructed not to count the dots and they were frequently reminded of this instruction.

If the child underestimated the position of the number of steps on the line, a feedback of a big elephant looking at the right appeared to indicate that the correct answer was located further on the right and he/she was allowed to try again until he/she succeeded. Similarly, if his/her response was an overestimation of the number of steps, a small mouse looking at the left appeared to indicate that he had to go less far on the line.

Both programs were adaptive; if the child had 85% correct answers on the last two thirds of the items of the current level, he/she moved up to the next level; otherwise he/she started the last two thirds of the trials again. In each session, the child played each game for 15 minutes.

After opening the laptop and settling each child with the game, the experimenter did not interact with him/her, except to encourage him/her to continue the game, as all instructions were given verbally and individually into headphones. Children did not interact with each other either.

**2. Control training:** Children in the control group were stimulated for the same amount of time by the experimenter with activities which did not involve any numerical aspect. In groups of five, they were told a story from a picture book and then asked questions about the story.

## Results

### Training Check

All training conditions (non-symbolic, symbolic and control) were successful in maintaining children’s motivation over the whole study period (see Table 1 for achieved levels details).

**Table 1:** Game level at the end of training for each training game in each group.

		Non-symbolic group	Symbolic group
Number line game	Level 1	5	0
	Level 2	9	1
	Level 3	5	13
	Level 4	0	4
	All levels achieved	0	1
Comparison game	Level 1	0	0
	Level 2	0	0
	Level 3	0	3
	Level 4	2	3
	Level 5	9	1
	Level 6	7	5
	All levels achieved	1	7

### Analyses

In the first section, we presented some preliminary analyses: comparison of the three groups in terms of age and pre-test performance and analysis of the reliability of the tasks. Then, to assess the effect of training, we conducted repeated-measures ANOVAs with Time (T1: pre-test and T2: post-test) as within-subjects factor and Group (non-symbolic, symbolic and control) as between-subjects factor on each of the testing tasks scores. When the interaction between the time and the group was significant, we computed contrasts with Bonferroni correction, to compare T1 and T2 performance for each group and calculated the partial Eta square as a measure of effect size. We also tested the validity of each task by adding difficulty level (ratio and condition (congruent and incongruent) for non-symbolic comparison, size for number lines and symbolic comparison, distance for symbolic comparison) as within-subjects factor. To keep the analysis section clear, these effects were reported only when they interacted with time and group, i.e., in the collection comparison and the non-symbolic number line tasks (see S1 Appendix for detailed analyses of the other tasks).

Given the fact that there is so much variability in performance with young children (due to fatigue, lack of attention, loss of motivation, etc.), for each task analysis, participants who obtained a score below 2 standard deviations compared to the mean of their group at T1 or T2 were excluded. This resulted in three outliers for the collection comparison task (1 in the non-symbolic and 2 in the symbolic group, performance range from 25% to 44%), four for the non-symbolic number line (2 in the symbolic and 2 in the control group, performance range: 31%-49%), three for the Arabic number comparison task (2 in the symbolic and 1 in the control group, 40%-69%), two for the verbal number comparison task (1 in the non-symbolic and 1 in the symbolic group, 48%-52%), five for the symbolic number line (2 in the non-symbolic, 1 in the symbolic and 2 in the control group, 25%-44%) and one for the addition task (symbolic group, 42%).

### Preliminary analyses

A one-way ANOVA indicated that the groups were statistically equivalent in age (non-symbolic group: 5.8 years old ± 4 months; symbolic group: 5.7 years old ± 4 months; control group: 5.10 years old ± 4 months) as well as in their performance (see Table 2) in all the testing tasks at T1 (collection comparison: *F*(2,52) = 1.81; *p* = .174; non-symbolic number line: *F*(2,51) < 1;Arabic number comparison: *F*(2,52) = 1.23; *p* = .302; verbal number comparison: *F*(2,53) < 1; symbolic number line: *F*(2,50) < 1; exact additions: *F*(2,53) = 1.4; *p* = .256).

**Table 2:** Groups’ mean and standard deviations for each variable at T1 and at T2 (%).

Variable	Non-symbolic group			Symbolic group			Control group		
	T1	T2	N	T1	T2	N	T1	T2	N
Collection comparison	49±23	72±8	18	60±18	68±10	17	57±15	61±18	18
Non-symbolic number line (PAE)	20±7	15±3	19	22±9	17±5	17	23±8	20±9	16
Arabic number comparison	79±15	85±13	19	85±12	90±8	17	85±11	88±9	17
Verbal number comparison	73±10	74±11	18	69±12	76±10	18	68±10	69±13	18
Symbolic number line (PAE)	16±6	14±4	17	18±5	13±3	18	17±7	17±6	16
Addition	54±15	61±17	18	62±16	76±14	18	65±19	65±21	18

Cronbach’s alphas were calculated to examine the reliability of our tasks (see Table 3).

**Table 3:** Cronbach’s alpha for each of the testing measures at T1 and T2.

Task	Cronbach’s alpha	
	T1	T2
Collection comparison	.82	.68
Non-symbolic number line	.77	.75
Arabic number comparison	.85	.81
Verbal number comparison	.69	.75
Symbolic number line	.72	.66
Exact additions	.41	.55

### Collection comparison

In addition to the factors time and group, we introduced ratio (1/2–2/3–3/4–4/5) as within-subjects factor.

**Fig 5:**
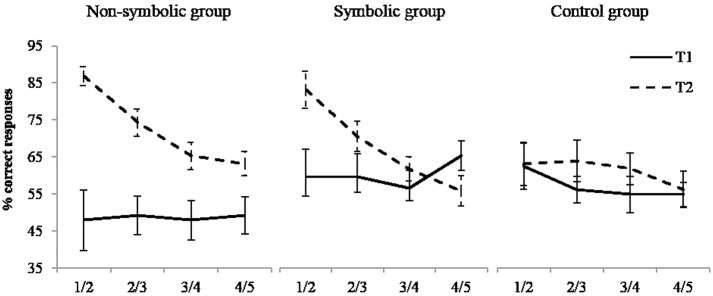
Performance in the collection comparison task. Illustration of the interaction between ratio and time in the non-symbolic, the symbolic and the control groups. Error bars represent standard errors.

The main effects of time, *F*(1,50) = 15.36; p = .002, η^2^ = 0.235, and ratio, *F*(3,150) = 7.85; *p* <.001, η^2^ = 0.136, were significant; performance increased from T1 (55±19%) to T2 (67±13%) and decreased with increasing ratio (means of 67±20% for 1:2; 62±15% for 2:3; 58±13% for 3:4; 57±12% for 4:5). There was no effect of group, *F*(1,50) = 0.80, *p* = 454, η^2^ = 0.031. More importantly, we found a significant interaction between time and group, *F*(2,50) = 4.09; *p* = .023, η^2^ = 0.140, ratio and time, *F*(3,150) = 8.68; *p* <.001, η^2^ = 0.148, and ratio, time and group, *F*(6,150) = 2.91, *p* = .016, η^2^ = 0.104; see Fig 5. The ratio x time interaction was significant in the two experimental groups (non-symbolic group: *F*(3,51) = 6.08; *p* = .001, η^2^ = 0.263, and symbolic group: *F*(3,48) = 6.25; *p* = .005, η^2^ = 0.281) but not in the control group, *F*(3,51) = 1.17, *p* = .331, η^2^ = 0.064. Corrected contrasts comparing performance at T1 and T2 for each ratio and each group showed that (1) in the non-symbolic group, performance increased from T1 to T2 for ratios 1:2, *t*(50) = 4.94; *p* <.001, η^2^ = 0.328 (means of 48±35% and 87±11%), 2:3, *t*(50) = 4; *p* <.001, η^2^ = 0.243 (means of 49±22% and 74±16%) and 3:4, *t*(50) = 2.90; *p* = .048, η^2^ = 0.144 (48±22% and 65±15%); (2) in the symbolic group, performance increased from T1 to T2 for the 1:2 ratio only, *t*(50) = 2.91; *p* = .040, η^2^ = 0.145 (60±31% and 83±21%).

To sum up, the non-symbolic group improved its performance for ratios 1:2, 2:3 and 3:4 and the symbolic group showed improvement for ratio 1:2, while there was no change of performance in the control group.

### Non-symbolic number line

**Fig 6:**
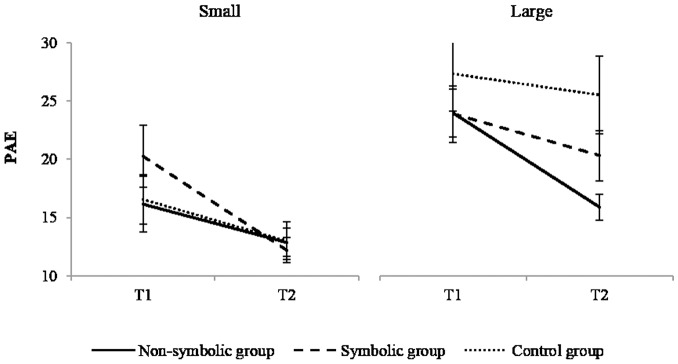
Performance in non-symbolic number line. Illustration of the decrease of PAE from T1 to T2 in each group for small and large numerosities. Error bars depict standard errors.

The analysis was computed with time, size (small (2–9) and large (10–19) items) and group as factors. The main effects of time, *F*(1,49) = 18.57; *p* <.001, η^2^ = 0.275, and of size, *F*(1,49) = 28.16, *p* <.001, η^2^ = 0.365, were significant; the PAE significantly decreased from T1 to T2 (22±8% and 17±6%) and small numbers (15±6%) were better positioned than large ones (23±9%). The effect of group, *F*(1,49) = 1.57, *p* = .219, η^2^ = 0.060 and the interaction effects between time and group, *F*(2,49) = 0.85, *p* = .433; η^2^ = 0.034, between size and group, *F*(2,49) = 1.87, *p* = .165, η^2^ = 0.071, and between time and size, *F*(1.49) = 0.09, *p* = .761, η^2^ = 0.002, were non-significant. In contrast, the effect of interaction between time, size and group was significant, *F*(2,49) = 3.25, *p* = .047, η^2^ = 117, indicating a significant decrease of PAE for large numerosities in the non-symbolic group, *t*(49) 3.91, *p* <.001, η^2^ = 0.238 (Fig 6), and for small numerosities in the symbolic group (Fig 6), *t*(49) = 3.22, *p* = .012, η^2^ = 0.174 (Table 4).

**Table 4:** Means ± standard deviations in the non-symbolic number line task.

Group	Small numbers		Large numbers	
	T1	T2	T1	T2
Non-symbolic	16±10%	13±5%	24±9%	16±5%
Symbolic	20±11%	12±4%	24±10%	24±9%
Control	17±9%	13±6%	27±13%	26±13%

Thus, the non-symbolic group improved on large numerosities and the symbolic group on small numerosities.

### Arabic number comparison task

**Fig 7:**
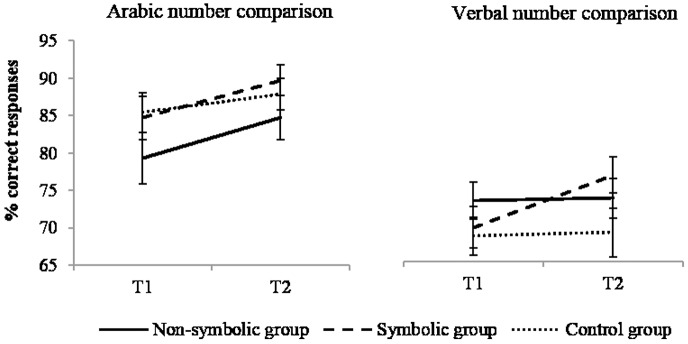
Performance in Arabic number and verbal number comparison tasks. Error bars represent standard errors.

The effect of time was significant, *F*(1,50) = 11.44; *p* = .001, η^2^ = 0.186, performance increased from T1 to T2 (83±13% and 88±12%, respectively) and the effect of group was non-significant, *F*(1,50) = 1.29, *p* = .286, η^2^ = 0.049. There was no effect of training, *F*(2,50) = 0.54, *p* = .585; η^2^ = 0.021 (Fig 7).

### Verbal number comparison task

The main effect of time was significant, *F*(1,51) = 5.49; *p* = .023, η^2^ = 0.097; performance increased from T1 (70±11%) to T2 (73±12%). The effect of group was non-significant, *F*(1,51) = 1.22, *p* = .305, η^2^ = 0.046. These effects marginally interacted with each other, *F*(2,51) = 2.94; *p* = .062, η^2^ = 0.103 (Fig 7). Considering the size of the sample, despite the marginality of this interaction, we decided to go further into the analyses and contrasts testing the difference in performance between T1 and T2 were computed for each group. They indicated a highly significant enhancement of performance for the symbolic group, *t*(51) = 3.33; *p* = .006, η^2^ = 0.178, but not for the non-symbolic, *t*(51) = 0.45; ns, η^2^ = 0.005, or control, *t*(51) = 0.23; ns, η^2^ = 0.001, groups.

Thus, the symbolic condition only led to significant improvement.

### Symbolic number line

**Fig 8:**
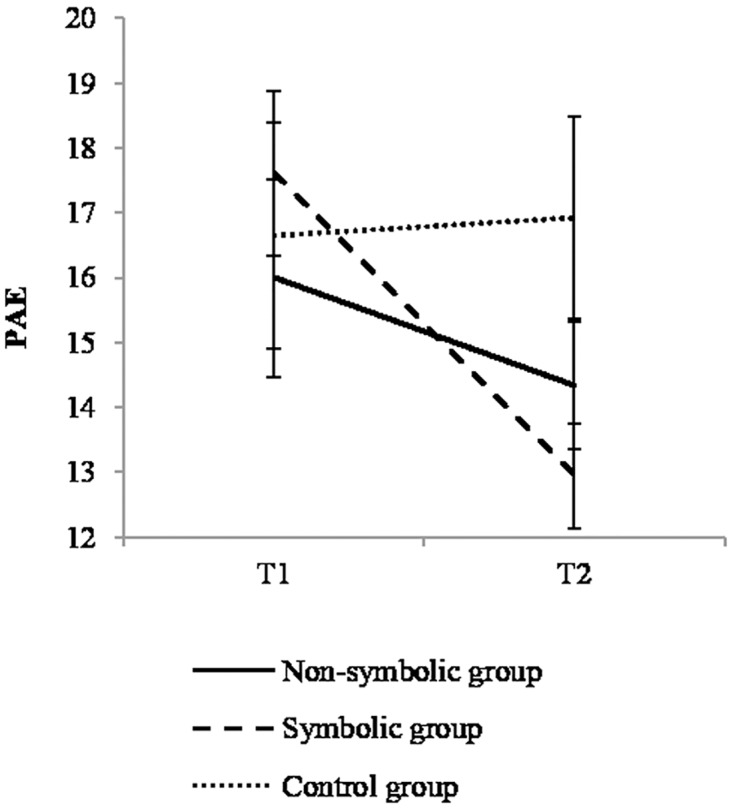
Performance in non-symbolic and symbolic number line. Error bars depict standard errors.

The main effect of time was significant, *F*(1,48) = 6.19; *p* = .016, η^2^ = 0.114; imprecision decreased from T1 to T2 (17±6% to 15±5%) and there was no effect of group, *F*(1,48) = 0.42, *p* = .662, η^2^ = 0.017. More importantly, the interaction effect between time and group was significant, *F*(2,48) = 3.31, *p* = .045, η^2^ = 0.121 and corrected contrasts revealed a significant decrease of imprecision in the symbolic group, *t*(48) = 3.45; *p* = .003, η^2^ = 0.198, only (*t*(48) = 1.23; *p* = .672, for the non-symbolic group, η^2^ = 0.031, and *t*(48) = 0.08, ns, η^2^ = 0 for the control group, see Fig 8).

The symbolic group showed a significant improvement, while the non-symbolic and the control groups did not.

### Exact arithmetic

**Fig 9:**
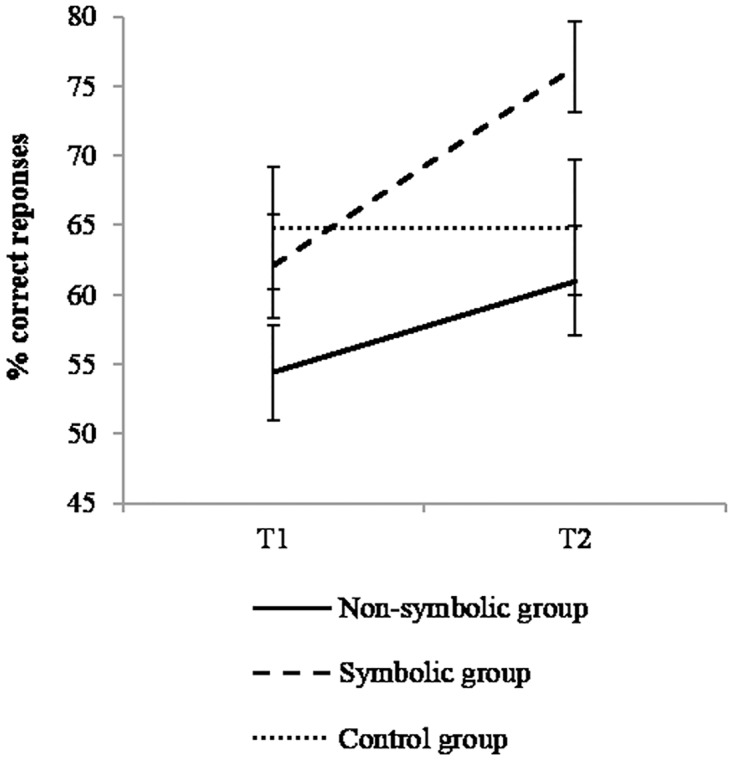
Performance in exact addition. Interaction between time and group. Error bars represent standard errors.

There was a significant effect of time, *F*(1,52) = 12.62, *p* = .001, η^2^ = 0.195; performance increased from T1 to T2 (6±17% and 67±18%) and the effect of group was non-significant, *F*(1,52) = 2.67, *p* = .079, η^2^ = 0.093. More importantly, the interaction between time and group was significant, *F*(2,52) = 4.38; *p* = .017, η^2^ = 0.144 (Fig 9). Improvement in performance over time was significant in the symbolic group only, *t*(52) = 4.18; p < .001, η^2^ = 0.265 (*t*(52) = 1.97, p = .166, η^2^ = 0.069 for the non-symbolic group, and *t*(52) = 0, ns, η^2^ = 0 for the control group).

A significant improvement was observed in the symbolic group only.

Finally Pearson’s correlation coefficients *r* were calculated as a measure of effect size, lying between 0 (no effect) and 1 (perfect effect) and calculated as follow: $\sqrt{\frac{F}{F+DF\;error}}$ [70]. The training effect sizes we have observed are important and comparable to or higher than the effects found in other studies (Table 5).

**Table 5:** Training effect sizes (r) in different training studies, including the present study.

Study	Collection comparison		Non-symbolic number line		Arabic number comparison	Verbal number comparison	Symbolic number line	Additions
Kucian et al. (2011) [63]							.66	.54
Link et al. (2013)’s embodied number line [64]					.22		.55	.55
Link et al. (2013)’s non embodied group [64]					.27			
Maertens et al. (2016)’s comparison group [62]								
Maertens et al. (2016)’s number line group [62]			.73				.74	
Obersteiner et al. (2012)’s approximate group [57]	.30				.18			
Sella et al. (2016) [59]	.31				.41			.71
Siegler and Ramani (2009)					.58		.67	.48
Vilette et al. (2013) [66]								.85
Wilson, Revkin et al. (2006) [61]	.74				.84			
Wilson et al. (2009)’s first group (Number Race then control) [60]					.57	.57		
Wilson et al. (2009)’s second group (control then Number Race) [60]					.32	.40		
**Present study—non-symbolic group**	**.74**		**All items**	**Large items**	**.52**			
			**.41**	**.70**				
**Present study—symbolic group**	**All ratios**	**Ratio 1/2**	**All items**	**small items**		**.64**	**.64**	**.72**
	**.33**	**.38**	**.40**	**.63)**				

## Discussion

The present study aimed to compare the differential effects of non-symbolic and symbolic training on numerical development and on arithmetic, and therefore to compare two existing views of numerical development. The unique representation view suggests that numerical and arithmetical competences rely on the ability to approximately represent the number magnitude of both symbolic and non-symbolic numerosities, through the ANS. In contrast, the dual-representation view considers that two distinct number magnitude representations underlie the processing of symbolic and non-symbolic numbers, one exact and one approximate. According to this view, the exact system is considered the basis of mathematical learning.

Our training programs differed in terms of format used. In the non-symbolic version of training, participants were presented with collections of dots; in the symbolic version, Arabic numbers were used. Except for the material used, the two versions of training were identical, enabling us to make comparisons. They were both composed of one comparison and one number line game. Although, the two training games rely on different mechanisms and are often used separately in the literature, they also share common underlying processes, as both of them are assumed to tap magnitude representation and have been shown to impact arithmetic performance [62]. In this study, they were used together to maximize the chance of improving number magnitude skills and minimize the possibility of boredom as young children need change to stay interested and motivated.

To take spontaneous development into account, we compared the performance of these two groups to a third control group, which received a story understanding stimulation.

First, the trained process was enhanced. Indeed, gains were observed after non-symbolic training in collection comparison and in the positioning of large non-symbolic numerosities on the number line and after symbolic training in the positioning of Arabic numbers on a number line. The absence of effect on Arabic number comparison could be due to the small size of our sample leading to low power of the analyses, which might explain why the increase of performance observed in the symbolic group was not significant. However, Maertens, De Smedt, Sasanguie, Elen, and Reynvoet [62] also failed to observe improvement in comparison tasks after magnitude comparison training and they suggest that this is due to a reduced malleability of comparison skills.

Second, a generalization to the auditory modality was observed in the symbolic group for the number words magnitude comparison task. This indicates that the training sessions helped children access the magnitude representation corresponding to symbolic numbers in general and not solely to the symbolic numbers presented in the Arabic format. We could thus hypothesize that training symbolic magnitude processing did in fact lead to the development of an exact representation of number magnitude.

Moreover, training symbolic number magnitude led to some improvement in non-symbolic magnitude processing: the comparison of collections (on 1/2 ratios) and the positioning of small collections of dots on a number line. The first result is in line with the finding that the learning of symbolic representations enhances the precision of the ANS [1, 54, 71]. Our results are also in line with Kolkman et al.’s study [30], which found that 5-year-old children’s non-symbolic skills were predicted by their symbolic skills at the age of 4, but not the reverse (i.e. 4-year-old non-symbolic skills did not predict symbolic skills at 5 years of age). The authors suggest that symbolic skills regulate the non-symbolic system by providing a meaning to non-symbolic magnitude and by improving mapping skills between the symbolic and non-symbolic quantities. The effect observed on the non-symbolic number line might result from a transfer of the symbolic-number-line enhancement. As all numerosities between 1 and 20 were to be positioned, the smallest ones fall in the subitizing range. Hence, participants might have subitized these small numerosities and used their improved ability to process symbolic numbers to position them.

Finally and more importantly, significant gains were observed in exact arithmetic after symbolic training only. Thus, training preschoolers in magnitude comparison and number line positioning with symbolic numbers, compared to non-symbolic numerosities, leads to greater improvement in calculation. This observation is important as, for the first time, it shows that specifically working on number magnitude has an impact on numerical development and arithmetic. Indeed, the few studies which showed improvement in mathematics not only trained basic number skills but also arithmetic as calculation problems were included in the training program [57, 58, 61, 63, 65, 66]. It could be argued that both training programs might not have been equally efficient. Nevertheless, although one limit of the present research is the impossibility of assessing the equivalence of the programs in terms of efficiency, the results indicated that they both led to improvement in the trained skills, which suggest that they did train what they were supposed to train. However, only symbolic training yielded improvement in the addition task. It could also be argued that mapping skills would come into play when performing this specific calculation task. However, it is a mapping between symbolic and non-symbolic numbers, which is very different from what has been trained, i.e. mapping from a number (Arabic number or a dot collection) onto space in the number line task.

Another limit of this study lies in the construction of the Arabic number comparison task, which seemed too easy. Indeed, 13 out of 56 participants performed higher than 95% at pre-test. As the number range was the same in the training and testing tasks, those high-achievers probably did not learn during training and this might have prevented sufficient variability in terms of gains. Increasing the difficulty of the training and testing Arabic number comparison tasks should thus be considered in future research.

Future research might also consider investigating the specific role of each training game, i.e. magnitude comparison and number line positioning, on numerical and arithmetical development, by contrasting their isolated training effect. In this line, Maertens et al. [62] have recently studied the differential impact of these two tasks used as training tools, using a mix of symbolic and non-symbolic numbers. They showed that both training tasks had a positive effect on arithmetic but they did not find any transfer from one task to another, suggesting that number comparison and number line estimation rely on different mechanisms.

Thus, taken altogether, our results are in line with the dual-representation view, according to which symbolic and non-symbolic processing rely on two distinct number magnitude representations; symbolic numbers activating an exact number representation and non-symbolic numerosities the ANS [22]. Indeed, the unique representation view anticipated a similar increase of performance in symbolic and non-symbolic magnitude processing after both symbolic and non-symbolic conditions, which is not what we observed. Training non-symbolic skills led to improvement in non-symbolic processing only whereas the symbolic condition yielded increase of performance in number magnitude processing not only with symbolic numbers but also, to a lesser extent, with non-symbolic numerosities. An improvement in calculation was observed in the symbolic group only, meaning that it is the ability to process symbolic numbers which provides access to arithmetic. Thus, in line with several earlier research studies [1, 30, 49, 72, 73], our results underline the key-role played by the understanding of symbolic numbers in numerical development, prior to formal education.

Most of the effects we observed are important and are either comparable or higher than the effects found in other studies. In addition, while the two experimental groups showed improvement in some of the testing tasks, no performance increase was observed in the control group, demonstrating the efficiency of our training program compared to spontaneous development. Finally, as the same percentage of children in each training condition came from each school, the differential progressions between the groups cannot be explained by any possible variation in the impact of school activities.

Our results contribute to the literature not only by disentangling the existing debate regarding the influence of symbolic and non-symbolic understanding on mathematical learning, but also by supporting a theoretical view of numerical cognition.

## Supporting Information

S1 Appendix Detailed analyses. (DOCX)

S1 Data Collection comparison data. (XLSX)

S2 Data Non-symbolic number line data. (XLSX)

S3 Data Arabic number comparison data. (XLSX)

S4 Data Verbal number comparison data. (XLSX)

S5 Data Symbolic number line data. (XLSX)

S6 Data Addition data. (XLSX)

## References

[1] Noël MP, Rousselle L. Developmental Changes in the Profiles of Dyscalculia: An Explanation Based on a Double Exact-and-Approximate Number Representation Model. Frontiers in human neuroscience. 2011;5:165 10.3389/fnhum.2011.00165 22203797 PMC3243900

[2] Bugden S, DeWind NK, Brannon EM. Using cognitive training studies to unravel the mechanisms by which the approximate number system supports symbolic math ability. Current Opinion in Behavioral Sciences. 2016;10:73–80. 10.1016/j.cobeha.2016.05.002 28439530 PMC5399542

[3] Feigenson L, Libertus ME, Halberda J. Links Between the Intuitive Sense of Number and Formal Mathematics Ability. Child development perspectives. 2013;7(2):74–9. 10.1111/cdep.12019 24443651 PMC3891767

[4] Izard V, Sann C, Spelke ES, Streri A. Newborn infants perceive abstract numbers. Proc Natl Acad Sci U S A. 2009;106(25):10382–5. 10.1073/pnas.0812142106 19520833 PMC2700913

[5] Libertus ME, Brannon EM. Behavioral and neural basis of number sense in infancy. Current Directions in Psychological Science. 2009;18(6):346–51. 10.1111/j.1467-8721.2009.01665.x 20419075 PMC2857350

[6] Lipton JS, Spelke ES. Origins of number sense: large-number discrimintation in human infants. Psychological Science. 2003;14(5):396–401. 12930467 10.1111/1467-9280.01453

[7] Xu F. Numerosity discrimination in infants: Evidence for two systems of representations. Cognition. 2003;89(1):B15–B25. 10.1016/s0010-0277(03)00050-7 12893126

[8] Dehaene S, Dehaene-Lambertz G, Cohen D. Abstract representations of numbers in the animal and human brain. Trends Neurosciences. 1998;21:355–61. 10.1016/s0166-2236(98)01263-6 9720604

[9] Hauser MD, Tsao F, Garcia P, Spelke ES. Evolutionary foundations of number: spontaneous representation of numerical magnitudes by cotton-top tamarins. Proceedings Biological sciences / The Royal Society. 2003;270(1523):1441–6. 10.1098/rspb.2003.2414 12965007 PMC1691404

[10] Barth H, Kanwisher N, Spelke E. The construction of large number representations in adults. Cognition. 2003;86:201–21. 12485738 10.1016/s0010-0277(02)00178-6

[11] Feigenson L, Dehaene S, Spelke E. Core systems of number. Trends in Cognitive Sciences. 2004;8(7):307–14. 10.1016/j.tics.2004.05.002 . 15242690

[12] Halberda J, Feigenson L. Developmental change in the acuity of the "Number Sense": The Approximate Number System in 3-, 4-, 5-, and 6-year-olds and adults. Developmental psychology. 2008;44(5):1457–65. 10.1037/a0012682 . 18793076

[13] Halberda J, Ly R, Wilmer JB, Naiman DQ, Germine L. Number sense across the lifespan as revealed by a massive Internet-based sample. Proceedings of the National Academy of Sciences of the United States of America. 2012;109(28):11116–20. 10.1073/pnas.1200196109 22733748 PMC3396479

[14] Xu F, Spelke ES. Large number discrimination in 6-month-old infants. Cognition. 2000;74:B1–B11. 10594312 10.1016/s0010-0277(99)00066-9

[15] Halberda J, Mazzocco MM, Feigenson L. Individual differences in non-verbal number acuity correlate with maths achievement. Nature. 2008;455(7213):665–8. 10.1038/nature07246 . 18776888

[16] Pica P, Lemer C, Izard V, Dehaene S. Exact and approximate arithmetic in an Amazonian indigene group. Science. 2004;306(5695):499–503. 10.1126/science.1102085 . 15486303

[17] Piazza M. Neurocognitive start-up tools for symbolic number representations. Trends Cogn Sci. 2010;14(12):542–51. 10.1016/j.tics.2010.09.008 . 21055996

[18] Gilmore CK, McCarthy SE, Spelke ES. Symbolic arithmetic knowledge without instruction. Nature. 2007;447(7144):589–91. 10.1038/nature05850 . 17538620

[19] Piazza M, Pinel P, Le Bihan D, Dehaene S. A magnitude code common to numerosities and number symbols in human intraparietal cortex. Neuron. 2007;53(2):293–305. 10.1016/j.neuron.2006.11.022 . 17224409

[20] Temple E, Posner MI. Brain mechanisms of quantity are similar in 5-year-old children and adults. Proceedings of the National Academy of Sciences of the United States of America. 1998;95:7836–41. 9636237 10.1073/pnas.95.13.7836 PMC22775

[21] Venkatraman V, Ansari D, Chee MW. Neural correlates of symbolic and non-symbolic arithmetic. Neuropsychologia. 2005;43(5):744–53. 10.1016/j.neuropsychologia.2004.08.005 . 15721187

[22] Sasanguie D, De Smedt B, Reynvoet B. Evidence for distinct magnitude systems for symbolic and non-symbolic number. Psychological research. 2015 10.1007/s00426-015-0734-1 . 26708496

[23] Barth H, La Mont K, Lipton J, Spelke ES. Abstract number and arithmetic in preschool children. Proceedings of the National Academy of Sciences of the United States of America. 2005;102(39):14116–21. 10.1073/pnas.0505512102 16172388 PMC1236560

[24] Mundy E, Gilmore CK. Children's mapping between symbolic and nonsymbolic representations of number. Journal of experimental child psychology. 2009;103(4):490–502. 10.1016/j.jecp.2009.02.003 . 19327782

[25] Mazzocco MM, Feigenson L, Halberda J. Impaired acuity of the approximate number system underlies mathematical learning disability (dyscalculia). Child development. 2011;82(4):1224–37. 10.1111/j.1467-8624.2011.01608.x . 21679173 PMC4411632

[26] Piazza M, Facoetti A, Trussardi AN, Berteletti I, Conte S, Lucangeli D, et al Developmental trajectory of number acuity reveals a severe impairment in developmental dyscalculia. Cognition. 2010;116(1):33–41. 10.1016/j.cognition.2010.03.012 . 20381023

[27] Wilson AJ, Dehaene S. Number sense and developmental dyscalculia. Human Behavior, Learning, and the Developing Brain: Atypical Development. 2007.

[28] Matejko AA, Ansari D. Trajectories of symbolic and non-symbolic magnitude processing in the first year of formal schooling. PloS one. 2016;11(3):e0149863 10.1371/journal.pone.0149863 26930195 PMC4773065

[29] Holloway ID, Ansari D. Mapping numerical magnitudes onto symbols: the numerical distance effect and individual differences in children's mathematics achievement. Journal of experimental child psychology. 2009;103(1):17–29. 10.1016/j.jecp.2008.04.001 . 18513738

[30] Kolkman ME, Kroesbergen EH, Leseman PPM. Early numerical development and the role of non-symbolic and symbolic skills. Learning and Instruction. 2013;25:95–103. 10.1016/j.learninstruc.2012.12.001

[31] Mussolin C, Mejias S, Noël MP. Symbolic and nonsymbolic number comparison in children with and without dyscalculia. Cognition. 2010;115(1):10–25. 10.1016/j.cognition.2009.10.006 . 20149355

[32] Sasanguie D, De Smedt B, Defever E, Reynvoet B. Association between basic numerical abilities and mathematics achievement. British Journal of Developmental Psychology. 2012;30(2):344–57. 10.1111/j.2044-835X.2011.02048.x . 22550952

[33] Sasanguie D, Defever E, Maertens B, Reynvoet B. The approximate number system is not predictive for symbolic number processing in kindergarteners. Quarterly journal of experimental psychology. 2014;67(2):271–80. 10.1080/17470218.2013.803581 . 23767979

[34] Lyons IM, Nuerk HC, Ansari D. Supplemental Material for Rethinking the Implications of Numerical Ratio Effects for Understanding the Development of Representational Precision and Numerical Processing Across Formats. Journal of Experimental Psychology: General. 2015;144(5):1021–35. 10.1037/xge0000094.supp 26168037

[35] Bulthé J, De Smedt B, Op de Beeck HP. Format-dependent representations of symbolic and non-symbolic numbers in the human cortex as revealed by multi-voxel pattern analyses. NeuroImage. 2014;87:311–22. 10.1016/j.neuroimage.2013.10.049 . 24201011

[36] Gebuis T, Cohen Kadosh R, Gevers W. Sensory-integration system rather than approximate number system underlies numerosity processing: A critical review. Acta psychologica. 2016;171:17–35. 10.1016/j.actpsy.2016.09.003 . 27640140

[37] Gebuis T, Reynvoet B. The interplay between nonsymbolic number and its continuous visual properties. Journal of experimental psychology General. 2012;141(4):642–8. 10.1037/a0026218 . 22082115

[38] Gilmore C, Attridge N, Clayton S, Cragg L, Johnson S, Marlow N, et al Individual differences in inhibitory control, not non-verbal number acuity, correlate with mathematics achievement. PloS one. 2013;8(6):e67374 10.1371/journal.pone.0067374.g001 23785521 PMC3681957

[39] Buckley PB, Gillman CB. Comparison of digits and dot patterns. Journal of Experimental Psychology. 1974;103(6):1131 4457588 10.1037/h0037361

[40] Vanbinst K, Ghesquière P, De Smedt B. Numerical magnitude representations and individual differences in children's arithmetic strategy use. Mind, Brain, and Education. 2012;6(3):129–36. 10.1111/j.1751-228X.2012.01148.x

[41] Carey S. Cognitive foundations of arithmetic: evolution and ontogenisis. Mind & Language. 2001;16(1):37–55.

[42] Carey S. Bootstrapping and the origin of concepts. Daedalus. 2004;133:59–68.

[43] Carey S. The Origin of Concepts development Osic, editor. Oxford: Oxford University Press; 2009.

[44] Crollen V, Seron X. Over-estimation in numerosity estimation tasks: more than an attentional bias? Acta psychologica. 2012;140(3):246–51. 10.1016/j.actpsy.2012.05.003 . 22683704

[45] Roggeman C, Verguts T, Fias W. Priming reveals differential coding of symbolic and non-symbolic quantities. Cognition. 2007;105(2):380–94. 10.1016/j.cognition.2006.10.004 . 17125760

[46] Gordon P. Numerical cognition without words: evidence from Amazonia. Science. 2004;306(5695):496–9. 10.1126/science.1094492 . 15319490

[47] Cirino PT. The interrelationships of mathematical precursors in kindergarten. Journal of experimental child psychology. 2011;108(4):713–33. 10.1016/j.jecp.2010.11.004 21194711 PMC3043138

[48] De Smedt B, Noël MP, Gilmore C, Ansari D. How do symbolic and non-symbolic numerical magnitude processing relate to individual differences in children's mathematics skills? A review of evidence from brain and behavior. Trends in Neuroscience and Education. 2013;2:48–55.

[49] Fazio LK, Bailey DH, Thompson CA, Siegler RS. Relations of different types of numerical magnitude representations to each other and to mathematics achievement. Journal of experimental child psychology. 2014;123:53–72. 10.1016/j.jecp.2014.01.013 . 24699178

[50] Lyons IM, Ansari D. Foundations of children's numerical and mathematical skills: the roles of symbolic and nonsymbolic representations of numerical magnitude. Advances in child development and behavior. 2015;48:93–116. 10.1016/bs.acdb.2014.11.003 . 25735942

[51] Schneider M, Beeres K, Coban L, Merz S, Susan Schmidt S, Stricker J, et al Associations of non-symbolic and symbolic numerical magnitude processing with mathematical competence: a meta-analysis. Developmental science. 2016 10.1111/desc.12372 . 26768176

[52] Rousselle L, Noël MP. Basic numerical skills in children with mathematics learning disabilities: a comparison of symbolic vs non-symbolic number magnitude processing. Cognition. 2007;102(3):361–95. 10.1016/j.cognition.2006.01.005 . 16488405

[53] Le Corre M, Carey S. One, two, three, four, nothing more: an investigation of the conceptual sources of the verbal counting principles. Cognition. 2007;105(2):395–438. 10.1016/j.cognition.2006.10.005 17208214 PMC3880652

[54] Piazza M, Pica P, Izard V, Spelke ES, Dehaene S. Education enhances the acuity of the nonverbal approximate number system. Psychological Science. 2013;24(6):1037–43. 10.1177/0956797612464057 . 23625879 PMC4648254

[55] Hyde DC, Khanum S, Spelke ES. Brief non-symbolic, approximate number practice enhances subsequent exact symbolic arithmetic in children. Cognition. 2014;131(1):92–107. 10.1016/j.cognition.2013.12.007 . 24462713 PMC4061922

[56] Praet M, Desoete A. Enhancing young children's arithmetic skills through non-intensive, computerised kindergarten interventions: A randomised controlled study. Teaching and Teacher Education. 2014;39:56–65. 10.1016/j.tate.2013.12.003

[57] Obersteiner A, Reiss K, Ufer S. How training on exact or approximate mental representations of number can enhance first-grade students’ basic number processing and arithmetic skills. Learning and Instruction. 2012 10.1016/j.learninstruc.2012.08.004

[58] Räsänen P, Salminen J, Wilson AJ, Aunio P, Dehaene S. Computer-assisted intervention for children with low numeracy skills. Cognitive Development. 2009;24(4):450–72. 10.1016/j.cogdev.2009.09.003

[59] Sella F, Tressoldi P, Lucangeli D, Zorzi M. Training numerical skills with the adaptive videogame “The Number Race”: A randomized controlled trial on preschoolers. Trends in Neuroscience and Education. 2016;5(1):20–9. 10.1016/j.tine.2016.02.002

[60] Wilson AJ, Dehaene S, Dubois O, Fayol M. Effects of an adaptive game intervention on accessing number sense in low-socioeconomic-status kindergarten children. Mind, Brain, and Education. 2009;3(4):224–34.

[61] Wilson AJ, Revkin SK, Cohen D, Cohen L, Dehaene S. An open trial assessment of "The Number Race", an adaptive computer game for remediation of dyscalculia. Behavioral and brain function. 2006;2:20 10.1186/1744-9081-2-20 16734906 PMC1523349

[62] Maertens B, De Smedt B, Sasanguie D, Elen J, Reynvoet B. Enhancing arithmetic in pre-schoolers with comparison or number line estimation training: Does it matter? Learning and Instruction. 2016;46:1–11. 10.1016/j.learninstruc.2016.08.004

[63] Kucian K, Grond U, Rotzer S, Henzi B, Schonmann C, Plangger F, et al Mental number line training in children with developmental dyscalculia. NeuroImage. 2011;57(3):782–95. 10.1016/j.neuroimage.2011.01.070 . 21295145

[64] Link T, Moeller K, Huber S, Fischer U, Nuerk HC. Walk the number line—An embodied training of numerical concepts. Trends in Neuroscience and Education. 2013;2(2):74–84. 10.1016/j.tine.2013.06.005

[65] Siegler RS, Ramani GB. Playing linear number board games—but not circular ones—improves low-income preschoolers’ numerical understanding. Journal of Educational Psychology. 2009;101(3):545–60. 10.1037/a0014239

[66] Vilette B, Mawart C, Rusinek S. L’outil « estimateur », la ligne numérique mentale et les habiletés arithmétiques. Pratiques Psychologiques. 2010;16(2):203–14. 10.1016/j.prps.2009.10.002

[67] Wilson AJ, Dehaene S, Pinel P, Revkin SK, Cohen L, Cohen D. Principles underlying the design of "The Number Race", an adaptive computer game for remediation of dyscalculia. Behavioral and Brain Functions. 2006;2:19 10.1186/1744-9081-2-19 16734905 PMC1550244

[68] Rousselle L, Dembour G, Noël MP. Magnitude representations in williams syndrome: differential acuity in time, space and number processing. PloS one. 2013;8(8):e72621 10.1371/journal.pone.0072621 24013906 PMC3755976

[69] Piazza M, Izard V. How humans count: numerosity and the parietal cortex. The neuroscientist. 2009;15(3):261–73. 10.1177/1073858409333073 19436075

[70] Field A. Discovering statistics using SPSS. 3rd ed London: Sage; 2009 856 p.

[71] Barth H, Starr A, Sullivan J. Children's mappings of large number words to numerosities. Cognitive Development. 2009;24(3):248–64. 10.1016/j.cogdev.2009.04.001

[72] De Smedt B, Gilmore CK. Defective number module or impaired access? Numerical magnitude processing in first graders with mathematical difficulties. Journal of experimental child psychology. 2011;108(2):278–92. 10.1016/j.jecp.2010.09.003 . 20974477

[73] Vanbinst K, Ansari D, Ghesquiere P, De Smedt B. Symbolic Numerical Magnitude Processing Is as Important to Arithmetic as Phonological Awareness Is to Reading. PloS one. 2016;11(3):e0151045 10.1371/journal.pone.0151045 26942935 PMC4778857

