# Supplementary material for: Improving Preschoolers’ Arithmetic through Number Magnitude Training: The Impact of Non-Symbolic and Symbolic Training
Source: PLoS One. 2016 Nov 22;11(11):e0166685. doi: 10.1371/journal.pone.0166685 (PMC5119778; doi:10.1371/journal.pone.0166685)
Supplement: S1 Appendix — (DOCX) [file pone.0166685.s001.docx]

*Appendix*

Detailed Analyses

**Arabic number comparison task**

We conducted a repeated-measures ANOVA on the percentage of correct responses, with time, size (small and large numbers) and distance (far and near) as within-subjects factors and the group as between-subjects factor. The effects of size, *F*(1,50) = 46.53, *p*<.001, η² = 0.482, and distance, *F*(1,50) = 21.16, *p*<.001, η² = 0.297, were significant; small numbers (91±8%) were better performed than large ones (79±16%) and far number (88±11%) were better compared than close ones (83±12%). These effects interacted with each other, *F*(1,50) = 5.73, *p*=.020, η² = 0.103; the effect of distance was significant for small, *t*(50) = 6.19, *p*<.001, η² = 0.434 (far = 95±7% and close = 88±11%), and not for large numbers, *t*(50) = 1.71, *p*=.186, η² = 0.055 (81±18% and 78±16%). Then, the effect of size significantly interacted with time, *F*(1,50) = 8.62, *p*=.005; η² = 147, and marginally with group, *F*(2,50) = 2.93, *p*=.062, η² = 0.105. The first interaction indicated that the effect of time was significant for large numbers, *t*(50) = 3.52, *p*=.002, η² = 0.208 (T1 = 76±19% and T2 = 86±5%), and not for small numbers, *t*(50) = 1.25, *p*=.270, η² = 0.44 (90±10% and 92±7%). The second interaction revealed a significant size effect in the two experimental groups, *t*(50) = 5.93, *p*<.001, η² = 0.413 for the non-symbolic group (small = 91±10% and large = 73±19%) and *t*(50) = 3.69, *p*=.002, η² = 0.214 for the symbolic group(93±6% and 82±14%), and not in the control group, *t*(50) = 2.30, *p*=.078, η² = 0.096 (90±7% and 83±13%). Finally, all other interactions (Distance x Group, Time x Size x Group, Time x Distance, Time x Distance x Group, Size x Distance x Group, Time x Size x Distance and Time x Size x Distance x Group) were non-significant (*Fs* < 1, except for Time x Distance interaction effect, *F*(1,50) = 1.15, *p*=.289, η² = 0.022.

**Verbal number comparison task**

A 3 (groups) x 2 (time) x 2 (size: small and large numbers) x 2 (distance: far and near) repeated-measures analysis on the percentage of correct responses was conducted. The effect of size was significant; performance was higher for small numbers than for large ones, *F*(1,51) = 218.19, *p*<.001, η² = 0.811 (83±10% and 61±13, respectively). This effect significantly interacted with distance, *F*(1,51) = 5.07, *p*=.029, η² = 0.090; the distance effect was non-significant for both small and large items but went in opposite direction (2% and -3%). No other effect was significant (*Fs* < 1 for the main effect of distance and the interaction effects between distance and group; size and group; time and size; time and distance; time, size and group; size, distance and group; time, size and distance; and time, size, distance and group; and *F*(2,51) = 2.03, *p*=.141, η² = 0.074 for the interaction between time, distance and group).

**Collection comparison task**

A repeated-measures ANOVA was computed on the percentage of correct responses, with time, ratio (1/2, 2/3, 3/4 and 4/5) and condition (congruent and incongruent) as within-subjects factors and the group as between-subjects factor. The effect of condition, *F*(1,50) = 30.99, *p*<.001, η² = 0.383, was significant; congruent items (71±22%) were better performed than incongruent ones (52±10%). This effect interacted with the effect of time, *F*(1,50) = 4.42, *p*=.041, η² = 0.081; performance on congruent items significantly improved, *t*(50) = 3.29, *p*=.004, η² = 0.178 (T1 = 62±35% and T2 = 80±24%), while the improvement on incongruent items did was non-significant, *t*(50) = 2.25, *p*=.058, η² = 0.092 (49±14% and 55±13%). The interaction effect between condition and ratio was marginal, *F*(3,150) = 2.61, *p*=.054, η² = 0.050, revealing a significant effect of ratio in the congruent, *t*(48) =2.27, *p*=.008, η² = 0.244, but not in the incongruent condition, *t*(48) =1.15, *p*=.560, η² = 0.076. All other effects were non-significant (*Fs* < 1 for the interactions between ratio and group; condition and group; ratio, condition and group; and time, ratio, condition and group; *F*(2,50) = 1.68, *p*=.197, η² =0.063 for Time x Condition x Group interaction; and *F*(3,50) = 1.11, *p*=.349, η² = 0.022).

**Symbolic number line**

The repeated-measures ANOVA testing the effects of the time, the size (small (2-9) and large (10-19) items) and the group revealed a significant effect of size, *F*(1,48) = 42.88, *p*<.001, η² = 0.472, with a higher precision for small than for large numbers. And this effect interacted with the group, *F*(2,48) = 3.38, *p*=.042, η² = 0.123; the size effect (large items – small items) was significantly higher for the control than for the non-symbolic, *p*=.033 (multiple comparisons with Bonferroni adjustment), and the symbolic group, *p*=.054; while the non-symbolic and the symbolic groups did not differ from each other (*p*=1, 6±5 for the non-symbolic group, 5±5 for the symbolic group and 11±7 for the control group). The interaction effects between time and size, *F*(1,48) = 0.01, *p*=.950, η² = 0, and between time, size and group, *F*(2,48) = 0.28, *p*=.759 , η² = 0.11, were non-significant.

**Exact arithmetic**

The repeated-measures ANOVA was run with time and difficulty level (small and large sums) as within-subjects factors and group as between-subjects factor. The results indicated that small additions were better solved than large ones, *F*(1,52) = 7.48, *p*=.009, η² = 0.126 (67±17% and 61±18%). This effect of difficulty level did not interact with the group, *F*(2,52) = 0.04, *p*=.962, η² = 0.001, nor with the time, *F*(1,52) = 0.73, *p*=.397, η² = 0.014. Then the interaction between time, difficulty level and group was also non-significant, *F*(2,52) = 1.14, *p*=.329, η² = 0.042.
